# Clinical and immunohistochemical study of melanotic neuroectodermal tumor of infancy in the maxilla

**DOI:** 10.1590/S1679-45082018RC4025

**Published:** 2018-05-03

**Authors:** Hellen Bandeira de Pontes Santos, Aníbal Henrique Barbosa de Luna, Pedro Everton Marques Goes, Alexander Tadeu Sverzut, Cassiano Francisco Weege Nonaka, Pollianna Muniz Alves

**Affiliations:** 1 Universidade Estadual da Paraíba, Campina Grande, PB, Brazil.; 2 Universidade Federal da Paraíba, João Pessoa, PB, Brazil.; 3 Universidade Estadual de Campinas, Piracicaba, SP, Brazil.

**Keywords:** Neuroectodermal tumor, melanotic/diagnosis, Maxillary neoplasms/diagnosis, Immunohistochemistry, Microscopy, Child, Case reports, Tumor neuroectodérmico melanótico/diagnóstico, Neoplasias maxilares/diagnóstico, Imuno-histoquímica, Microscopia, Criança, Relatos de casos

## Abstract

Melanotic neuroectodermal tumor of infancy is a rare and fast-growing neoplasm. In this study, we describe the case of a 6-month-old female patient, who presented swelling in the anterior maxilla. Tomographic reconstruction showed an unilocular hypodense and expansive area associated with the upper right central primary incisor. The presumptive diagnoses were dentigerous cyst, adenomatoid odontogenic tumor, melanotic neuroectodermal tumor of infancy and rhabdomyosarcoma, and an incisional biopsy was performed. Microscopically, the lesion revealed a biphasic cell population, consisting of small, ovoid, neuroblastic-like cells and epithelioid cells containing melanin. Immunohistochemically, the melanocyte-like component was strongly and diffusely positive for HMB-45 and Melan-A, but weakly positive for S100. Based on these findings, definitive diagnosis of melanotic neuroectodermal tumor of infancy was established. Then, enucleation of the lesion was performed by careful curettage. After 2 year follow-up, no clinical or radiographical evidence of recurrence was verified. The present case highlights the importance of early diagnosis and therapeutic intervention at the appropriate time to achieve a favorable outcome for the patient.

## RESUMO

O tumor neuroectodérmico melanocítico da infância é uma neoplasia rara e de crescimento rápido. Neste estudo, relata-se o caso de uma paciente do sexo feminino de 6 meses de idade, que apresentou tumefação na região anterior de maxila. A reconstrução tomográfica revelou área unilocular hipodensa e expansiva associada ao incisivo central superior direito decíduo. Realizou-se biópsia incisional, considerando as hipóteses diagnósticas de cisto dentígero, tumor odontogênico adenomatoide, tumor neuroectodérmico melanocítico da infância e rabdomiossarcoma. Microscopicamente, a lesão revelou população celular bifásica, consistindo de células pequenas, ovoides, de aparência neuroblástica, e de células epitelioides, contendo melanina. A análise imuno-histoquímica demonstrou que o componente celular contendo melanina era positivo de forma intensa e difusa para HMB-45 e Melan-A, mas levemente positivo para S100. Com base nestes achados, foi estabelecido o diagnóstico definitivo de tumor neuroectodérmico melanocítico da infância. Em seguida, foi realizada a enucleação da lesão com curetagem cuidadosa. Após 2 anos de acompanhamento, não foram verificadas evidências clínicas ou radiográficas de recorrência. O presente caso destaca a importância do diagnóstico precoce e da intervenção terapêutica no momento apropriado, a fim de alcançar um desfecho favorável para o paciente.

## INTRODUCTION

Melanotic neuroectodermal tumor of infancy (MNTI) is a very rare melanin-containing neoplasm, usually diagnosed during the first year of life.^(^ ^1^ ^–^ ^4^ ^)^ Approximately 314 cases have been reported in the gnathic bones.^(^ ^4^ ^)^ The discussion about the origin of this tumor had led authors to use a variety of nomenclatures, such as congenital melanocarcinoma, retinal anlage tumor, pigmented congenital epulis, or melanotic progonoma.^(^ ^1^ ^,^ ^4^ ^)^ However, neural crest origin has been considered, as shown by immunohistochemical, ultrastructural, and cell culture studies.^(^ ^1^ ^,^ ^2^ ^,^ ^4^ ^)^

Despite being classically a benign tumor, in most cases, the MNTI grows rapidly, presents locally destructive invasion, and can cause deformities in adjacent structures.^(^ ^1^ ^–^ ^3^ ^)^ Furthermore, a recurrence rate of about 60% has been reported for these tumors, some undergo malignant transformation, and 5% to 10% produce metastases.^(^ ^1^ ^,^ ^3^ ^)^ Thus, early diagnosis minimizes the difficulties and risks associated with treatment, favoring an optimal outcome and normal infant development.^(^ ^3^ ^)^ We report a case of MNTI, emphasizing its clinical, imaging, histological and immunohistochemical characteristics.

## CASE REPORT

**Figure 1:**
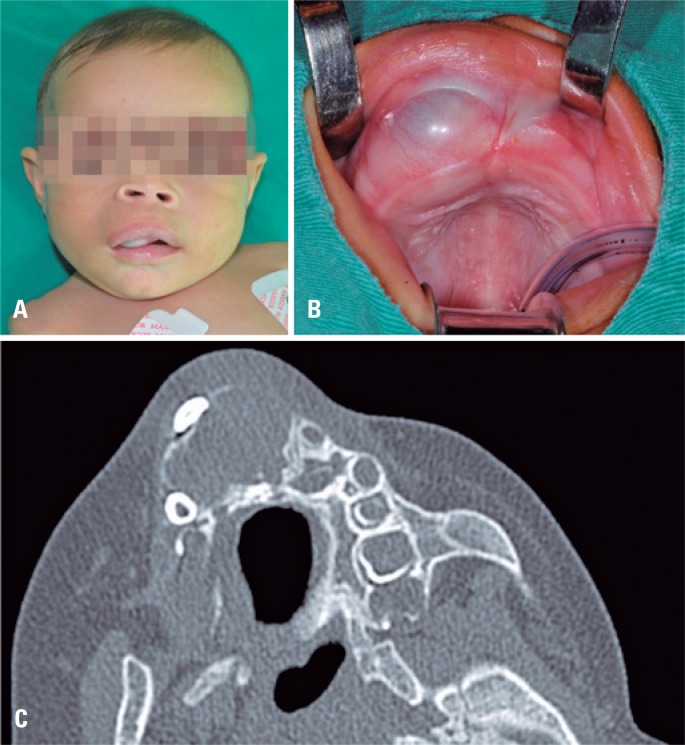
Clinical and imaginological findings of the patient. (A) Extraoral examination showing elevation of right upper lip and paranasal region. (B) Intraoral aspect of the melanotic neuroectodermal tumor of infancy revealing an expansive mass covered by intact mucosa. (C) Computed tomography scan showing a well-defined unilocular, osteolytic lesion causing expansion and destruction of the buccal cortical bone and involvement of the primary deciduous incisive tooth

A 6-month-old girl presented with swelling of the maxillary anterior alveolar ridge for 4 weeks, causing feeding difficulties. The patient had no congenital anomalies and no other abnormal physical or clinical findings. Extraoral examination showed superior displacement of the paranasal region and of the right upper lip (Figure 1A). Intraoral examination revealed a firm, reddish-bluish mass measuring approximately 4cm, extending from the right alveolar ridge to the hard palate, covered by an intact mucosa (Figure 1B). Needle aspiration produced negative results. The computed tomography scan showed a well-circumscribed osteolytic expansive mass in the right anterior maxilla, associated with the primary maxillary right central incisor (Figure 1C).

**Figure 2:**
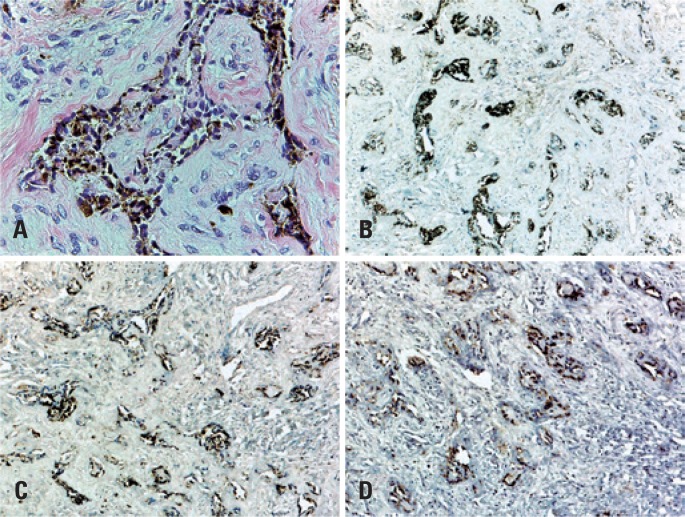
Histological and immunohistochemical aspects. (A) Islands of admixed pigmented, epithelial-like cells and alveolar aggregates of round cells in a fibrous connective tissue (H-E, 400×). (B) Epithelioid components were strongly positive for HMB-45 (100×). (C) Melan A diffusely positive in epithelial-like cells (100×). (D) Weak positivity for S100 in epitheloid components

The diagnoses of dentigerous cyst, adenomatoid odontogenic tumor, MNTI or rhabdomyosarcoma were suggested based on the clinical and imaging findings. For the definitive diagnosis, the patient underwent an incisional biopsy in the operating room. Microscopically, the lesion showed biphasic proliferation of small rounded neuroblast-like cells, and epithelioid cells with eosinophilic cytoplasm containing variable amounts of melanin (Figure 2A). No features of malignancy were observed. Immunohistochemically, the melanocyte-like component was strongly and diffusely positive for HMB-45 and Melan A (Figures 2B and 2C), and weakly positive for S100 (Figure 2D). The neuroblast-like component was not reactive to these antibodies.

**Figure 3:**
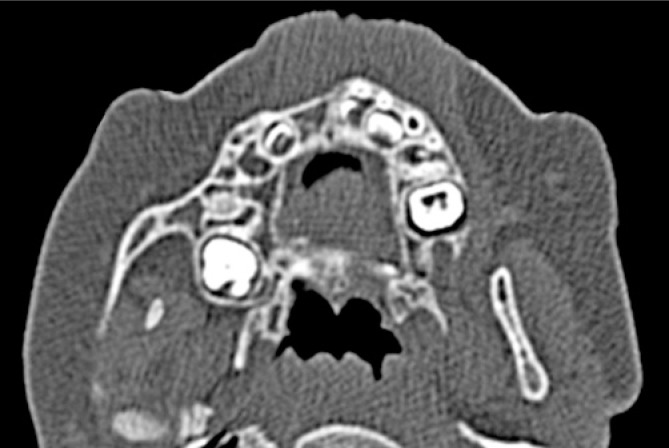
Axial computed tomography scan showing bone neoformation in the maxilla and no evidence of tumor recurrence

Based on these findings, the histopathological diagnosis was of MNTI. Thereafter, the lesion was easily enucleated and the bony cavity was carefully curetted and washed. The primary (deciduous) incisor was extracted since it was displaced buccally and had no osseous support. No postoperative morbidity was observed. Microscopic examination of the surgical specimen confirmed the diagnosis of MNTI. There has been no evidence of recurrence after 2 years (Figure 3).

## DISCUSSION

Melanotic neuroectodermal tumor of infancy is a rare, fast growing, benign tumor originating from the neural crest that usually occurs during the first year of life.^(^ ^1^ ^–^ ^4^ ^)^ In a comprehensive systematic review of 472 MNTI cases, from 1918 to 2013, Rachidi et al.,^(^ ^4^ ^)^ found that most cases occurred in the head and neck region, quite frequently in the maxilla (62.2%), skull (15.6%), and mandible (7.8%). To date, approximately 314 cases in the gnathic bones were reported in the literature. Moreover, half of the patients were younger than 4.5 months, and a slight male predominance was reported (56%).^(^ ^4^ ^)^

Clinically, as seen in the present case, MNTI appears as a painless, expansive, lobulated, partly pigmented tumor mass.^(^ ^1^ ^,^ ^2^ ^)^ Although well defined, it is usually an unencapsulated lesion that primarily affects the jaws of newborn children, frequently causing bone destruction and displacement of dental follicles.^(^ ^1^ ^,^ ^2^ ^)^ Some patients have high levels of urinary vanillylmandelic acid, which supports the neural crest origin of the tumor.^(^ ^4^ ^,^ ^5^ ^)^

Radiographic exams, such as computed tomography, can contribute to diagnosis, in addition to providing relevant information for surgical planning.^(^ ^3^ ^,^ ^5^ ^)^ Intraosseous MNTI lesions are commonly characterized by a well-circumscribed hypodense mass, and advanced-stage tumors show excessive bone destruction.^(^ ^5^ ^,^ ^6^ ^)^ The present case showed a well-defined, unilocular, osteolytic lesion causing expansion and destruction of the maxillary cortical bone. The differential diagnosis of MNTI affecting the head and neck region includes Ewing's sarcoma, lymphoma, odontogenic lesions, developmental cysts, rhabdomyosarcoma, metastatic neuroblastoma, infection, and nonodontogenic lesions, such as fibromatosis and fibrous dysplasia.^(^ ^4^ ^,^ ^5^ ^)^ Due to the wide variability in imaging results for MNTI, a tissue biopsy is required for the correct diagnosis.^(^ ^5^ ^)^

Histologically, MNTI are composed of small rounded neuroblast-like cellular areas, and of areas with large, polygonal, melanin-containing cells that combine neural, melanocytic, and epithelial cell types.^(^ ^4^ ^)^ In the present case, these microscopic features were found both in the incisional biopsy and in the surgical specimen. This heterogeneous cellular phenotype is probably explained by the mesodermal and ectodermal morphological features displayed by neural crest cells at different stages of their ontogeny.^(^ ^4^ ^)^ A recent study by Strieder et al.,^(^ ^7^ ^)^ investigated intratumoral immune cells of two MNTI cases using immunohistochemistry, and suggested involvement of M2-polarized macrophages in MNTI pathogenesis. In one case, dendritic-like cells positive for HLA-DR, XIIIa, CD68, and CD163 were observed in the fibrous septa and glia-like tissue, and in the tumor stroma in the other case. For the authors, these cells may act by modulating tumor growth and/or tumor stroma remodeling.

Melanotic neuroectodermal tumor of infancy may share a common histological and immunophenotypic expression with other lesions, such as cellular blue nevus, melanoma, neuroblastoma, and rhabdomyosarcoma, but MNTI commonly does not express diffuse reactivity with S-100,^(^ ^2^ ^,^ ^3^ ^,^ ^6^ ^,^ ^8^ ^)^ as observed in our case. Other markers, such as HMB45, Melan A, cytokeratin, and neuroblastic markers, such as synaptophysin and neuron-specific enolase, can help with the diagnosis.^(^ ^1^ ^,^ ^4^ ^)^ In the current case, melanin-producing epithelial cells were strongly positive for HMB-45 and Melan A. These findings are supported by Barrett et al.,^(^ ^1^ ^)^ Cui et al.,^(^ ^3^ ^)^ and Krishnamurthy et al.^(^ ^9^ ^)^

Generally, wide resection with 5-mm free margins and removal of the involved teeth are the treatment of choice for MNTI lesions.^(^ ^4^ ^,^ ^9^ ^)^ Chemotherapy alone and radiation therapy, either alone or in combination with chemotherapy, and resection have also been proposed.^(^ ^2^ ^,^ ^4^ ^)^ However, in the present case, the lesion was removed by enucleation and curettage. This treatment modality is curative when tumors are easily detached from the bone, as in this case.^(^ ^8^ ^)^ Moreover, esthetic sequelae and alteration in normal face development may be prevented by this conservative surgical approach.^(^ ^10^ ^)^ Rachidi et al.,^(^ ^4^ ^)^ reported that recurrence usually occurs within 6 months after treatment and in patients younger than 4.5 months. In our case, the patient had an uneventful postoperative course, and after 2 year follow-up, there has been no clinical or radiographic evidence of recurrence.

Due to the rapid growth of MNTI and its ability to cause major deformities in surrounding tissues, our case highlights the importance of early diagnosis, providing patients with a favorable and functional outcome. Thus, physicians, dentists, and other professionals should be aware of this tumor and refer patients to correct treatment in order to minimize mutilating surgeries.
